# Analysis of recurrent urinary tract infection management in women seen in outpatient settings reveals opportunities for antibiotic stewardship interventions

**DOI:** 10.1017/ash.2021.224

**Published:** 2022-01-17

**Authors:** Marissa A. Valentine-King, Barbara W. Trautner, Roger J. Zoorob, George Germanos, Michael Hansen, Jason L. Salemi, Kalpana Gupta, Larissa Grigoryan

**Affiliations:** 1 Department of Family and Community Medicine, Baylor College of Medicine, Houston, Texas; 2 Section of Health Services Research, Department of Medicine, Baylor College of Medicine, Houston, Texas; 3 Department of Surgery, Baylor College of Medicine, Houston, Texas; 4 Center for Innovations in Quality, Effectiveness and Safety (IQuESt), Michael E. DeBakey Veterans Affairs Medical Center, Houston, Texas; 5 Department of Internal Medicine, Baylor Scott and White Health, Round Rock, Texas; 6 College of Public Health, University of South Florida, Tampa, Florida; 7 VA Boston Healthcare System, Boston, Massachusetts; 8 Boston University School of Medicine, Boston, Massachusetts

## Abstract

**Objectives:**

We characterized antibiotic prescribing patterns and management practices among recurrent urinary tract infection (rUTI) patients, and we identified factors associated with lack of guideline adherence to antibiotic choice, duration of treatment, and urine cultures obtained. We hypothesized that prior resistance to nitrofurantoin or trimethoprim–sulfamethoxazole (TMP-SMX), shorter intervals between rUTIs, and more frequent rUTIs would be associated with fluoroquinolone or β-lactam prescribing, or longer duration of therapy.

**Methods:**

This study was a retrospective database study of adult women with *International Classification of Diseases, Tenth Revision* (ICD-10) cystitis codes meeting American Urological Association rUTI criteria at outpatient clinics within our academic medical center between 2016 and 2018. We excluded patients with ICD-10 codes indicative of complicated UTI or pyelonephritis. Generalized estimating equations were used for risk-factor analysis.

**Results:**

Among 214 patients with 566 visits, 61.5% of prescriptions comprised first-line agents of nitrofurantoin (39.7%) and TMP-SMX (21.5%), followed by second-line choices of fluoroquinolones (27.2%) and β-lactams (11%). Most fluoroquinolone prescriptions (86.7%), TMP-SMX prescriptions (72.2%), and nitrofurantoin prescriptions (60.2%) exceeded the guideline-recommended duration. Approximately half of visits lacked a urine culture. Receiving care through urology via telephone was associated with receiving a β-lactam (adjusted odds ratio [aOR], 6.34; 95% confidence interval [CI], 2.58–15.56) or fluoroquinolone (OR, 2.28; 95% CI, 1.07–4.86). Having >2 rUTIs during the study period and seeking care from a urology practice (RR, 1.28, 95% CI, 1.15–1.44) were associated with longer antibiotic duration.

**Conclusions:**

We found low guideline concordance for antibiotic choice, duration of therapy and cultures obtained among rUTI patients. These factors represent new targets for outpatient antibiotic stewardship interventions.

Urinary tract infections (UTIs) are a common condition in ambulatory care, accounting for an estimated 10.5 million visits to primary care annually.^1^ A subset of patients experience recurrent UTIs (rUTIs). Prospective studies identified UTI recurrence in 24% of college-aged females within 6 months and in 44% of adult women (mean age, 48 years) within 1 year.^2,3^ Sequalae include physical pain and negative impacts on physical and social functioning, which are further amplified in patients with rUTI.^4^

Evidenced-based rUTI management recommendations in the *British Medical Journal* in 2013 and the 2018 American Urogynecologic Society (AUS) best-practice statement specific to rUTI management in females largely follow the 2011 Infectious Disease Society of America (IDSA) guidelines for uncomplicated cystitis in terms of antibiotic selection and duration.^5,6^ After accounting for availability, allergy history, tolerance, and regional resistance, recommended agents available in the United States include nitrofurantoin, trimethoprim-sulfamethoxazole (TMP-SMX), and fosfomycin, prescribed for 5-, 3-, and 1-day periods, respectively.^7^ Fluoroquinolones and β-lactams are considered second-line agents. Due to potentially serious side effects, the US Food and Drug Administration (FDA) issued an initial ‘black box’ warning in 2008 for fluoroquinolones. In 2016, the FDA recommended fluoroquinolone use only in uncomplicated UTI (uUTI) patients with no alternative treatment.^8,9^ The AUS statement and others also recommend obtaining a urine culture prior to antibiotic initiation, prescribing vaginal estrogen therapy in peri- or postmenopausal females, and providing low-dose antibiotic prophylaxis.^5,6,10^

Multiple studies have investigated prescribing practices for sporadic, uUTI in ambulatory settings and found substantial discordance with IDSA guidelines in terms of antibiotic choice and duration.^11–14^ Durkin et al^12^ found overall IDSA guideline concordance at 26% in terms of antibiotic choice, dose, and duration.^12^ Among 6 family medicine practices, Cowart et al^11^ found that >75% of prescriptions exceeded the recommended treatment duration. Overall, fluoroquinolone prescribing prevalence in uUTI ranged from 35.3% to 51.6% across 4 studies.^11–14^

To the best of our knowledge, no US studies have examined prescribing or management practices among patients with rUTI—a population at greater risk for experiencing adverse events from repeated antibiotic exposure.^15^ Therefore, our study objectives included characterizing antibiotic choice and duration, nonantibiotic UTI-related therapies, and the percentage of urine cultures obtained at visits. We also identified factors associated with guideline nonadherence in terms of antibiotic choice, duration of therapy, and lacking a visit-associated urine culture. We hypothesized that antibiotic resistance to a first-line agent, a shorter duration between visits and more frequent rUTIs may trigger providers to select a second-line agent or extend therapy duration in the latter 2 cases.

## Materials and methods

### Setting and study design

We conducted a retrospective database study of adult females who sought care for uncomplicated rUTI between November 1, 2016, and December 31, 2018, at family medicine, internal medicine, and urology clinics within an academic medical center in a large urban area. The patient population at these clinics generally includes privately insured patients; however, public insurance (Medicare or Medicaid) is also accepted.^14^ Patients do not require a referral for primary care or internal medicine practices. However, a referral to urology may be required depending on insurance plans. In general, referrals to urology are sought when a patient presents with any of the following: elevated prostate-specific antigen test, hematuria, suspected anatomic abnormality, or noteworthy voiding difficulties. Noninfectious disease primary care physicians typically refer patients with rUTI to urology or infectious disease for care.

Data procurement occurred over 2 stages: (1) electronic extraction of patient data from the Epic Clarity Database that met our inclusion and exclusion criteria based on *International Classification of Diseases Tenth Revision* (ICD-10) codes; and (2) a manual chart review to identify additional ICD-10 codes and visit details indicating complicated UTI or pyelonephritis.

### Inclusion and exclusion criteria

**Fig. 1.**
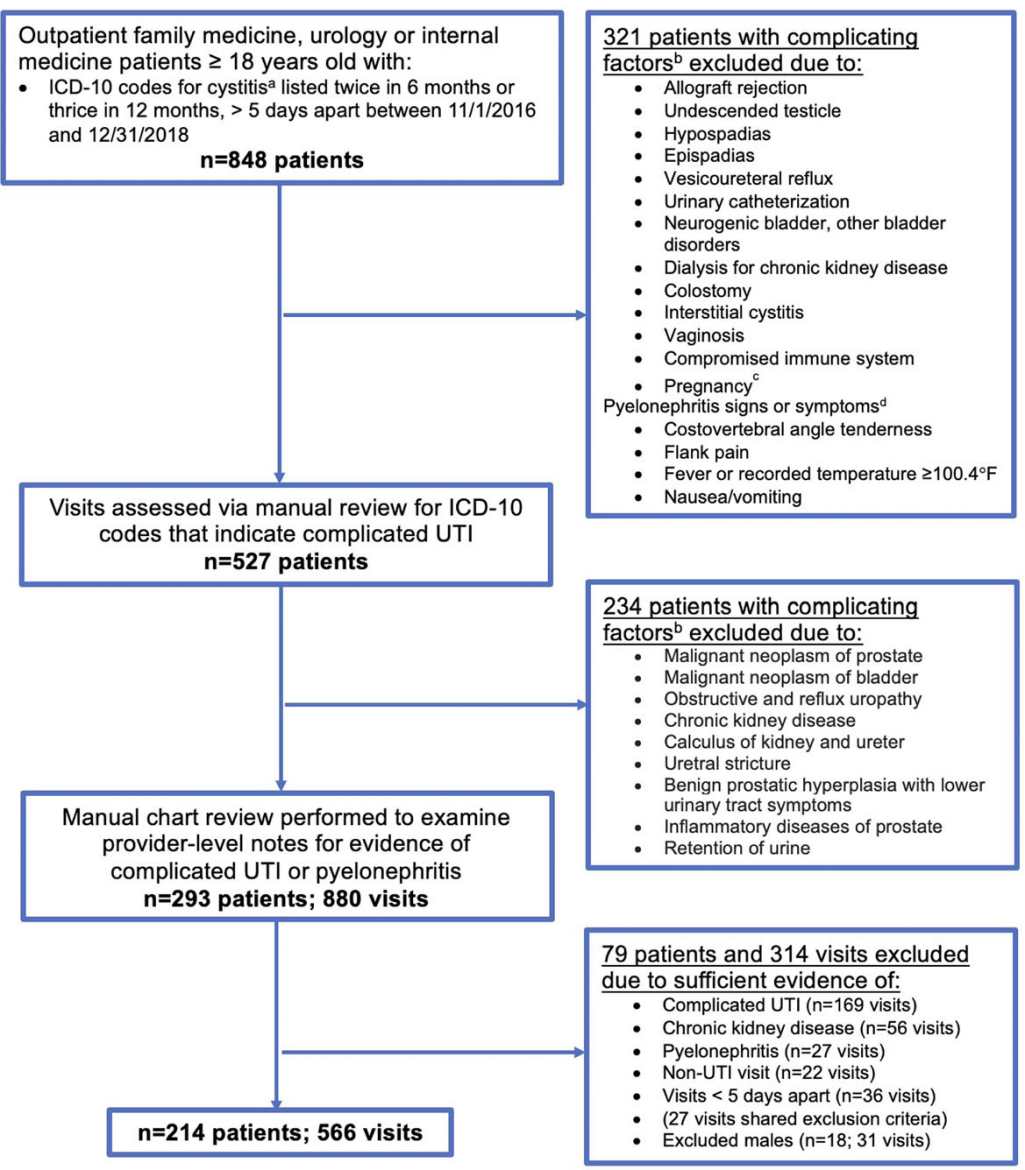
Flow chart depicting methods for rUTI patient and visit inclusion and exclusion process. ^a^*International Classification of Diseases* (ICD-10) codes N30.0, N30.9, and N39.0. ^b^Exclusionary ICD-10 codes for complicating factors were applied for all visits 12 months prior or at the qualifying visit. Exclusionary codes included T86, Q53, Q54, Q64.0, N13.7, T83, N31, N32, Z99.2, Z93.3, N30.10, N30.11, N76.0, D89.9, Z33.1, Z33.3 C61, C67, N13, N18, N20, N35, N40, N41 and R33. ^c^Excluded if the ICD-10 code listed in the prior year or 6 months after the qualifying visit. ^d^Exclusionary criteria for pyelonephritis only applied at qualifying visit; ICD-10 codes were R50.9 and R11.

Figure 1 provides details on the selection process and criteria for study inclusion and exclusion. Females 18 and older were included if their record contained an ICD-10 code for cystitis (N30.0, N30.9, and N39.0) that occurred either in 2 visits within 6-months or 3 visits within 12 months (qualifying events), per the American Urological Association definition for rUTI.^10^ Visits also had to occur >5 days apart to exclude any follow-up visits, and records had to be available 6 months following the last visit to capture recurrences within 12 months. Patients were excluded if they had ICD-10 codes listed in the prior year or at their qualifying visit indicating complicating factors that would impact the structural integrity or functional ability of the genitourinary tract, interstitial cystitis, vaginosis, impaired immune functioning, or an ICD-10 code for fever or nausea (qualifying visit only).

During the manual chart review, a trained clinician reviewed the patient history, review of symptoms, physical exam, and provider assessment to identify and exclude cases of pyelonephritis (Fig. 1).^16^ The problem list, history of present illness, and differential diagnosis were examined to identify and exclude patients with chronic kidney disease or complicated UTI. Sufficient evidence of complicated UTI included a urologic abnormality, evidence indicating an immunocompromised state (eg, HIV, corticosteroid use, autoimmune disease), pregnancy, and nephrolithiasis.

For each eligible visit, we extracted demographic details and the following visit data: encounter date, type (office or telephone), practice type, ICD-10 codes, and allergy data. Antibiotic type, frequency, and quantity, coupled with urine culture data including organism type, concentration and antibiotic susceptibility were extracted when present. We searched and extracted urine culture results within a 3-day window before and after the visit date. Elixhauser scores were calculated for each visit by identifying ICD-10 codes matching any Elixhauser comorbidity category and assigning points based on a weighted metric validated by Moore et al. for predicting hospital readmission.^17^ In addition, patients with clinical evidence of diabetes mellitus, based on HbA1c scores, diabetic medications, and a history of diabetes mellitus, were assigned the corresponding points for having ‘uncomplicated diabetes’ per Moore et al. and were also evaluated independently. The Baylor College of Medicine Ethics Committee approved the study protocol.

### Statistical analysis

Descriptive statistics were calculated for demographic, health, and visit data, nonantibiotic therapies, and antibiotic type and duration. Antibiotics were classified as prophylactic if therapy exceeded 14 days or were directed to use ‘as needed.’ We used the 2011 IDSA recommended duration of therapy for uUTI of 7 days for β-lactams, 5 days for nitrofurantoin, and 3 days for fluoroquinolones and TMP-SMX when evaluating whether duration exceeded guideline recommendations.^7^ When multiple antibiotics were listed for the same visit, these were counted independently in the overall antibiotic summary. Patients prescribed a second-line agent with allergies to both nitrofurantoin and TMP-SMX (5 patients, 8 visits) were excluded from the descriptive totals and risk factor analysis for antibiotic choice.

To identify factors associated with antibiotic choice for episodic therapy, we used generalized estimating equations (GEE) with a logit link (multivariate logistic regression). For antibiotic choice, we evaluated factors associated with prescription of a second-line agent (β-lactam or fluoroquinolone) compared to a first-line agent (nitrofurantoin, TMP-SMX or fosfomycin). Visits in which both first- and second-line agents were prescribed were excluded.

We used GEE Poisson regression to evaluate predictors associated with lacking a visit-associated urine culture and duration of antibiotic therapy. We included all antibiotics prescribed episodically except for ceftriaxone and doxycycline, as the former was a one-time, intravenous administration, while the latter serves as a non-traditional antibiotic for UTI. If a visit contained different antibiotics, each was included separately in the analysis.

GEE analyses were conducted in R Studio version 1.5.17 software (R Foundation for Statistical Computing, Vienna, Austria) using the *geepack* package specifying an exchangeable correlational structure.^18^ A backward, stepwise regression process was used with a threshold *P* < .20 required to enter the model and 0.1 to stay in the multivariable model. We evaluated collinearity between significant predictors in the univariate analysis using GEE. A *P* < .05 was considered significant. We detected collinearity between practice type and visit type because telephone visits were significantly associated with urology (*P* < .001). Therefore, we combined both practice and visit type into a multilevel categorical variable (Table 4). This was also done for urine culture ordering, but with internal medicine separated from family medicine. More frequent rUTI visits and urology were also significantly associated (*P* < .001) and subcomponents were parsed out (Table 5).

## Results

The rUTI population consisted of 214 unique patients that had 566 visits. The majority were white (63.1%) females that had a median age of 56 (IQR, 40–58) (Table 1). Approximately 19% of patients had evidence of diabetes mellitus; however, the median Elixhauser score was 0 (IQR, 0–0), indicating a relatively healthy population. Patients sought care for UTI a median of 2 times (IQR, 2–3), and two-thirds of those visits transpired in an office setting and one-third via the telephone. Slightly more than half of the rUTI population received care at internal or family medicine practices compared to urology (46%).

**Table 1.** Patient Characteristics and Recurrent UTI Visit Details

Patient Characteristics	Cohort (n=214), No. (%)
Age, median (IQR)^a^	56 (40–68)
**Race**	
White	135 (63.1)
Black	27 (12.6)
Other^b^	52 (24.3)
**Marital status**	
Single	62 (29)
Married or significant other	109 (50.9)
Divorced/separated/widowed	32 (15)
Other/unknown	11 (5.1)
**Language spoken**	
English	205 (95.8)
Spanish	8 (3.7)
Other	1 (0.5)
Diabetes mellitus	40 (18.7)
Elixhauser score, median (IQR)	0 (0–0)
**Visit details**	**(n=566)**
Visits per patient, median (IQR)	2 (2–3)
Interval between visits, median d (IQR)	58 (22–122)
**Visit type**	
Office visit	378 (66.8)
Telephone	188 (33.2)
**Practice type**	
Family medicine	264 (46.6)
General internal medicine	41 (7.2)
Urology	261 (46.1)
**Symptoms reported** ^**c**^	
Dysuria	196 (73.7)
Urgency	164 (75.6)
Frequency	178 (79.5)
Hematuria	39 (26.5)
Incontinence	28 (68.3)

Note. UTI, urinary tract infection; IQR, interquartile range.

a Age from first rUTI visit in study period.

b Other includes Asian (n=10), American Indian (n=1), Hispanic (n=19), and unknown (n=22).

c The percentage of reported symptoms was calculated based on visits that had explicit documentation of the patient’s symptoms (either as present or absent).

### Antibiotic choice details

Overall, 61.5% of patients received a first-line agent and 27.2% had a fluoroquinolone prescribed for episodic rUTI (Table 2). Nitrofurantoin was the most common first-line drug prescribed (39.7%) followed by TMP-SMX (21.5%), while fosfomycin was prescribed only once. β-lactams comprised 11% of prescribed antibiotics and one patient received an intravenous ceftriaxone dose in addition to oral antibiotics. Prophylactic therapy was more aligned with rUTI treatment recommendations, with 93.2% of antibiotics falling within a recommended category.

**Table 2.** Descriptive Summary of Antibiotics Prescribed to Patients With Recurrent UTI by Episodic and Prophylactic Treatment

Antibiotics Prescribed^a^	Total (n=395)	Episodic (n=335)^d^	Prophylactic (n=44)^e^
**β-lactams**	59 (14.9)	37 (11)	3 (6.8)
Amoxicillin	7 (1.8)	4 (1.2)	3 (6.8)
Amoxicillin-clavulanate	13 (3.3)^c^	11 (3.3)	…
Ampicillin	1 (0.3)	1 (0.3)	…
Cefpodoxime	3 (0.8)^c^	1 (0.3)	…
Ceftriaxone	1 (0.3)	1 (0.3)	…
Cefuroxime	11 (2.8)	11 (3.3)	…
Cephalexin^b^	23 (5.8)^c^	8 (2.4)	NA
**Fluroquinolones**	95 (24.1)	91 (27.2)	…
Ciprofloxacin	84 (21.3)^c^	81 (24.2)	…
Levofloxacin	11 (2.8)^c^	10 (3)	…
**First-line treatments**	240 (60.8)	206 (61.5)	41 (93.2)
Fosfomycin	1 (0.3)	1 (0.3)	…
Nitrofurantoin	163 (41.3)^c^	133 (39.7)	25 (56.8)
TMP-SMX	76 (19.2)^c^	72 (21.5)	2 (4.6)
Cephalexin^b^	NA	NA	14 (31.8)
Other	1 (0.3)	1(0.3)	
Doxycycline	1 (0.3)	1 (0.3)	…

Note. NA, not applicable.

a Excluded second-line antibiotics prescribed to patients with allergies to first-line agents (nitrofurantoin and TMP-SMX).

b Cephalexin is considered a first-line therapy for prophylactic treatment; therefore, in column 4, it is included as part of ‘first-line treatments’ and excluded from ‘β-lactams.’

c Contains observations with an unknown length of treatment.

d Episodic antibiotic therapy defined as treatment duration ≤14 d.

e Prophylactic therapy defined as antibiotic duration >14 d.

### Antibiotic duration details

**Fig. 2.**
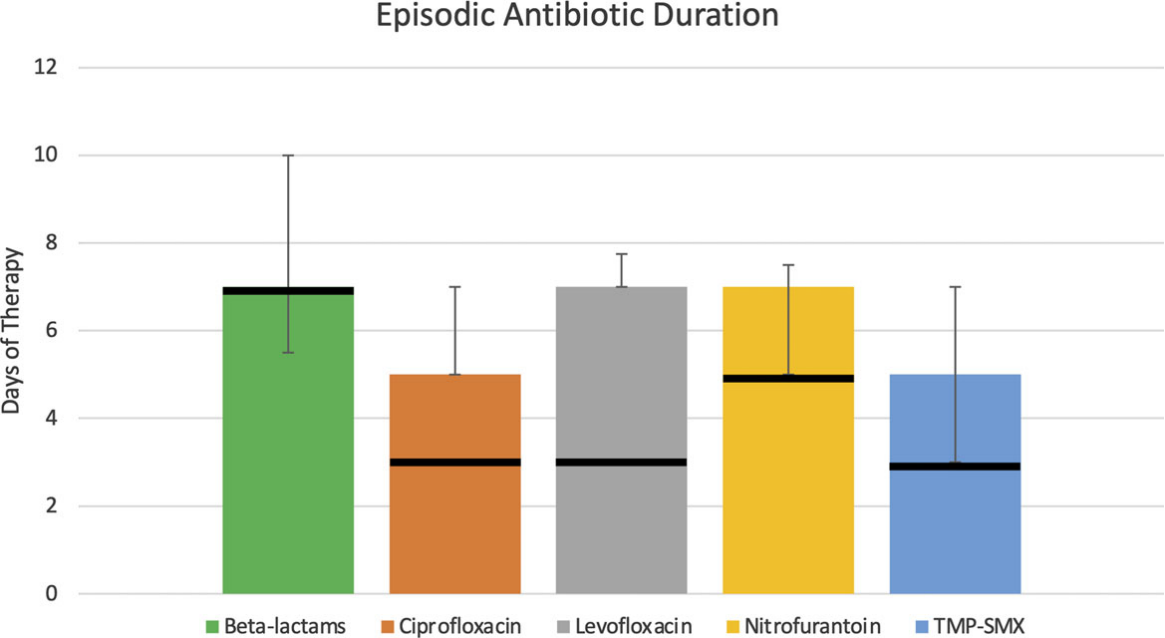
Episodic antibiotic duration—a bar chart depicting the median length of therapy by antibiotic class or drug for episodic rUTI treatment. Error bars represent first and third quartiles, and solid black lines represent recommended duration of therapy according to the Infectious Disease Society of America guidelines for uncomplicated cystitis.^7^

Duration of therapy also exceeded IDSA guideline recommendations, with the median duration eclipsing the 3-day cutoff points for fluoroquinolones and TMP-SMX, and 5-day threshold for nitrofurantoin (Fig. 2). Ciprofloxacin, levofloxacin, TMP-SMX, and nitrofurantoin prescriptions had inappropriate durations in 85.2%, 100%, 72.2%, and 60.2% of cases, respectively.

### Nonantibiotic therapies

Nonantibiotic therapies were prescribed to a lesser extent. Only 11.7% of eligible females had vaginal estrogen therapy prescribed. Other agents less frequently prescribed included nonopioid pain relievers (7.2%), bladder antispasmodics (3.2%), cranberry products, and probiotics (0.9%) (Supplementary Table 1).

### Urine cultures obtained and predictors

Only 52% of visits had an accompanying urine culture. In a sensitivity analysis evaluating a patient’s second visit or later, only 44.8% of visits had a culture. Visit-associated cultures were highest for office visits (66.4%) but were lower among telephone visits (22.9%). Urine cultures collected also varied by practice type: internal medicine had the lowest percentage (34.1%), followed by urology (42.5%). Also, 64% of family medicine visits had a documented culture. Regression modeling found seeking care at internal medicine, regardless of visit type, increased the risk of lacking a urine culture; however, telephone visits across all practice types displayed higher relative risks compared to office encounters (Table 3). In the univariate analysis, patients within ∼2 months of their prior visit had a 26% higher risk of not having a urine culture; however, this association became nonsignificant in the multivariate model (*P* = .074).

**Table 3.** Factors Associated With Lacking a Visit-Associated Urine Culture Using Generalized Estimating Equations Poisson Regression

Predictors	Urine Culture Ordering			
	Univariate Model		Multivariate Model^a^	
	RR (95% CI)	*P* Value	aRR (95% CI)	*P* Value
Age	**1 (1–1.01)**	**.17**	…	…
Elixhauser score	…	.73	…	…
**Race**				
White	Reference	…	Reference	…
Black	…	.86	…	…
Other	…	.64	…	…
**Practice and visit type**				
FM office	Reference	…	Reference	…
FM telephone	**2.99 (2.27–3.94)**	**<.001**	**2.38 (1.73–3.26)**	**<.001**
IM office	**2.24 (1.45–3.45)**	**<.001**	**2.09 (1.35–3.24)**	**.001**
IM telephone	**2.89 (1.98–4.20)**	**<.001**	**2.43 (1.72–3.43)**	**<.001**
Urology office	**1.55 (1.13–2.13)**	**.007**	1.43 (0.99–2.07)	.055
Urology telephone	**2.95 (2.32–3.76)**	**<.001**	**2.58 (1.97–3.38)**	**<.001**
**Interval between UTI** ^**a**^				
>56 d	Reference	…	Reference	…
≤56 d	**1.26 (1.02–1.54)**	**.03**	1.18 (0.98–1.43)	.074
No. of UTI visits	…	.25	…	…
Antibiotic resistance in prior culture^b,c^	…	.97	…	…

Note. aRR, adjusted relative risk; FM, family medicine; IM, internal medicine.

Bold text indicates a significant finding, or a finding with a *P*-value <0.05.

a Different sample size (n=362) compared to overall sample size (n=566) for this variable/model.

b Defined as having a urine culture on the previous visit with resistance to either nitrofurantoin or TMP-SMX.

c Different sample size (n=111) compared to overall sample size (n=566) for this variable.

### Predictors of second-line therapies

Telephone visits with urology significantly increased the odds of β-lactam and fluoroquinolone prescribing by 6.34 and 2.28 times, respectively, but urology office visits did not (Table 4). In the univariate analysis, primary-care telephone visits were also associated with β-lactam prescribing but became nonsignificant in the final model. Contrary to our expectations, we did not find significant associations between prior resistance to a first-line agent, decreased intervals between UTIs or more frequent UTIs, and prescribing second-line agents.

**Table 4.** Factors Associated With β-Lactam or Fluoroquinolone Prescribing, Using Generalized Estimating Equations Logistic Regression

Predictors	β-Lactam Prescribing				Fluoroquinolone Prescribing	
	Univariate Model		Multivariate Model		Univariate Model	
	OR (95% CI)	*P* Value	aOR (95% CI)	*P* Value	OR (95% CI)	*P* Value
Age	**1.03 (1.01–1.05)**	**.006**	1.02 (1.00–1.04)	.115^e^	…	.49
**Race**						
White	Reference	…	Reference	…	Reference	…
Black	0.86 (0.22–3.42)	.83	…	…	…	.797
Other	**0.47 (0.16–1.38)**	**.17**	…	…	…	.225
Elixhauser score	…	.830	…	…	…	.38
Diabetes mellitus	…	.84	…	…	…	.43
**Practice and visit type**						
Primary care^a^ office	Reference	…	Reference	…	Reference	…
Primary care telephone	**2.87 (1.01–8.15)**	**.047**	2.74 (0.94–7.95)	.064	0.94 (0.42–2.08)	.876
Urology office	**3.09 (0.71–13.47)**	**.13**	2.41 (0.44–13.10)	.309	0.94 (0.34–2.61)	.898
Urology telephone	**7.84 (3.18–19.32)**	**<.001**	**6.34 (2.58–15.56)**	**<.001**	**2.28 (1.07–4.86)**	**.033**
Interval between UTI, d^b^	**1 (0.99–1.00)**	**.100**	…	…	…	.67
No. of UTI visits	**1.08 (1.01–1.14)**	**.014**	…	…	…	.58
Antibiotic resistance in prior culture^c,d^	**2.63 (0.71–9.67)**	**.146**	…	…	…	.60

Note. aOR, adjusted odds ratio; CI, confidence interval.

Bold text in the univariate model indicates a *P*-value < .2 and inclusion in the initial model. Bold text in the multivariate model indicates a significant finding, or a finding with a *P*-value <0.05.

a Includes patients seeking care at family medicine or internal medicine.

b Different sample size for β-lactams (n=134)/fluoroquinolones (n=153) compared to overall sample size for β-lactams (n=233)/ fluoroquinolones (n=284) for this variable.

c Defined as having a urine culture on the previous visit with resistance to either nitrofurantoin or TMP-SMX.

d Different sample size for β-lactams(n=45)/fluoroquinolones (n=54) compared to overall sample size for β-lactams (n=233)/ fluoroquinolones (n=284) for this variable.

e Age included in final β-lactam model due to its role as a confounder.

### Predictors of antibiotic duration

Having 3 or more visits with urology increased therapy days by 28% (95% CI, 1.15–1.44), but this was not the case for primary care patients with 3 or more visits (OR, 1.09; 95% CI, 0.99–1.19) or urology patients with 2 visits (OR, 1.11; 95% CI, 0.94–1.33) (Table 5). We found no evidence to support our hypothesis that a shorter interval between visits (OR, 1; 95% CI, 1–1) was associated with longer duration of therapy.

**Table 5.** Factors Associated With Antibiotic^a^ Duration in Days Using Generalized Estimating Equations Poisson Regression

Predictors	Antibiotic Duration in Days	
	Univariate Model	
	RR (95%CI)	*P* Value
Age	1 (1–1)	.21
**Race**		
White	Reference	…
Black	0.92 (0.81–1.04)	.20
Other	1.029 (0.93–1.14)	.57
Elixhauser score	…	.21
Clinical evidence of DM	…	.74
**Practice type and number of visit**		
Primary care^b^ 2 visits	Reference	…
Primary care > 2 visits	1.09 (0.99–1.19)	.068
Urology 2 visits	1.11 (0.94–1.33)	.22
Urology >2 visits	**1.28 (1.15–1.44)**	**<.001**
**Visit type**		
Office visit	Reference	…
Telephone	…	.72
Diabetes mellitus	…	.74
Interval between UTI (days)^c^	…	.27
Prior resistant culture^d,e^	…	.95

Note. RR, relative risk; CI, confidence interval; aOR, adjusted relative risk; DM, diabetes mellitus.

Bold text indicates a significant finding, or a finding with a *P*-value <0.05.

a Includes β-lactams, fluoroquinolones, nitrofurantoin, and TMP-SMX, while excluding ceftriaxone (n=1). When visits contained duplicate entries for same antibiotic (n=10), only 1 instance was used for the analysis.

b Includes patients seeking care at family medicine or internal medicine practice.

c Different sample size (n=192) compared to the overall sample size (n=330), as no interval available for first visit.

d Defined as having a urine culture on the previous visit with resistance to either nitrofurantoin or TMP-SMX.

e Different sample size (n=66) compared to overall sample size (n=330); not each visit had a prior visit with susceptibility data.

## Discussion

Our study revealed moderate concordance (61.5%) with first-line agent prescribing; however, fluoroquinolones were prescribed in 27% of visits and almost 90% exceeded the 3-day recommended duration. Overall, only 21.5% of prescriptions consisted of a first-line agent prescribed for the guideline-concordant duration. Lack of concordance with choice of drug and duration of therapy increases opportunities for antibiotic resistance and adverse drug events. For example, Chalmers et al^19^ found each additional day of antibiotics increased the risk of *C. difficile* infection by 9% and a meta-analysis found antibiotic courses >3 days significantly increased the risk of adverse drug reactions by 17%.^19,20^ Meanwhile, patients with trimethoprim courses >7 days had 2.89 higher odds of developing resistance than those with regimens <7 days (95% CI, 1.44–5.78).^19–21^

In terms of management, only 52% of visits had an accompanying urine culture result. The 2018 AUS best-practice statement and others advocate for urine culture ordering to establish susceptibility information and confirm rUTI diagnosis.^5,6,10^ The uropathogen sensitivity data are key to ensure antibiotics with congruent susceptibility profiles are selected, especially with heightened levels of antibiotic resistance from selective pressure induced by repeated antibiotic therapy. Two recent studies detected uropathogen-antibiotic susceptibility mismatch in 31% and 40% of patients presenting at emergency departments (EDs) for a UTI.^22,23^ In one of the studies, uropathogen-cephalexin susceptibility mismatch significantly increased the odds of ED readmission.^22^ This finding underscores the potential for drug–pathogen susceptibility mismatches, which can delay appropriate treatment with the targeted drug, increase healthcare utilization, and associated costs.

A positive urine culture helps confirm a UTI, and a negative culture should prompt consideration of other diagnoses. In a prospective study, 22% of catheterized and mid-stream urine samples from adult females symptomatic for UTI had no uropathogen growth (0 colony forming units (CFU)/mL).^24^ Tomas et al^25^ analyzed urine cultures from ED patients diagnosed with UTI and found that only 48% had a positive culture (≥10^3^ CFU/mL). In addition, 37% of those with sexually transmitted infections were misdiagnosed as UTIs.^25^ Thus, a negative urine culture could facilitate treatment of the underlying cause and prevent the patient from receiving inappropriate antibiotics.

Factors associated with lacking a urine culture included seeking care at internal medicine or having telephone visits at urology or family medicine practices. Having a telephone visit compared to an office visit in each setting further increased the risk of lacking a culture by an additional 238%, 34%, and 115% in family medicine, internal medicine, and urology clinics, respectively. Ewen et al^26^ examined telephone prescribing practices in ambulatory settings and found the most common indication for antibiotic prescribing was for UTI, and >75% of antibiotics were prescribed empirically without a culture, which supports our findings. Murray et al^27^ found significantly lower levels of urine culture ordering for episodic UTI in an RN-led telephone treatment protocol (7%), compared to face-to-face visits (21%). However, the relationship between decreased urine cultures and practice type is not clear.

Telephone visits with urology were significantly associated with having a β-lactam or fluoroquinolone ordered. Ewen et al^26^ found telephone visits compared to office visits had significantly higher levels of fluoroquinolones prescribed overall, and fluoroquinolones were most frequently prescribed for telephone based-UTI treatment. Interestingly, herein, the relationship between telephone visits and prescribing second-line agents only demonstrated significance in urology. Urology practices may attract patients that suffer from rUTI over longer periods, and providers may opt for a second-line agent if a first-line agent fails to provide long-term resolution. Furthermore, patients with a longer history of rUTI may opt to call clinicians rather than schedule an office visit.

In terms of antibiotic duration, patients with more frequent rUTIs that sought care at a urology practice had significantly longer therapy duration. Urology practices may attract patients that experience rUTIs over longer periods of time. This, coupled with more frequent rUTIs may spur providers to extend therapy duration to reverse that patient’s disease course. Thus, the confluence of these factors may prompt providers to extend therapy in this subset of patients; urology patients with 2 rUTIs or primary care patients with >2 rUTIs did not have significantly longer therapy.

Very few women received vaginal estrogen therapy, even though it can significantly lower rUTI recurrence.^5,6^ Estrogen plays myriad of beneficial roles in promoting eubiotic effects that hamper colonization with uropathogenic bacteria.^28–30^ Thus, incorporating vaginal estrogen therapy into practice may be a target for stewardship as an antibiotic-sparing means to prevent rUTI among perimenopausal and postmenopausal women.

Although our findings stem from a single academic medical center, we considered 4 clinics representing 3 different medical fields: family medicine, internal medicine, and urology. Use of an electronic health record database can be prone to diagnosis coding inconsistences or incomplete charting. Incomplete ICD-10 coding may have created information bias because the extent of comorbidities may have been underreported, potentially underestimating Elixhauser scores and obscuring their relationship with prescribing outcomes. We mitigated these effects by conducting a manual chart review to examine the problem list and medical history for evidence of complicated UTI or chronic kidney disease. We also captured the presence of UTI symptoms, lowering the probability of including patients with asymptomatic bacteriuria; however, explicit affirmation or denial of symptoms were not available for all visits. Our urine culture variable depends not only on a written order but also the patient fulfilling the request. Thus, this outcome may reflect shortcomings in terms of ordering and in a patient’s inability or failure to submit a specimen. Medications captured only represented orders and medications listed from that specific visit. Therefore, nonantibiotic therapies may have been underrepresented if ordered through a different provider. Lastly, we did not capture other factors that may contribute to prescribing practices, such as provider type (MD vs DO vs NP) or years in practice, due to anonymity.

In conclusion, we conducted the first examination of rUTI prescribing practices in a US outpatient setting. Fewer than 25% of prescriptions consisted of a first-line agent at the guideline-recommended duration. We detected suboptimal levels of visit-associated urine cultures, which could lead to delayed or missed treatment for other comorbidities that mimic UTI symptomology or to uropathogen antibiotic-susceptibility mismatch. Telephone encounters across all practice types were associated with decreased urine cultures obtained, and telephone visits with urology were associated with both β-lactam and fluoroquinolone prescribing. Thus, these settings and visit types are potential stewardship targets. Resistance or allergies to first-line agents did not explain second-line agent selection, indicating that these prescribing practices can change. Overall, our study has demonstrated opportunities to further antibiotic stewardship and to improve management among rUTI patients.

## Supplementary material

For supplementary material accompanying this paper visit https://doi.org/10.1017/ash.2021.224. click here to view supplementary material
