# Supplementary material for: Analysis of recurrent urinary tract infection management in women seen in outpatient settings reveals opportunities for antibiotic stewardship interventions
Source: Antimicrob Steward Healthc Epidemiol. 2022 Jan 17;2(1):e8. doi: 10.1017/ash.2021.224 (PMC9614978; doi:10.1017/ash.2021.224)
Supplement: Supplementary file 1 [file S2732494X21002242sup001.docx]

**Supplemental Table 1.** Non-antibiotic therapies

| **Therapy Type^a^** | **No. (%)** |
| --- | --- |
| Anti-spasmodics^b^ | 15 (3.2) |
| Cranberry products | 4 (0.9) |
| Non-opioid pain-relievers | 34 (7.2) |
| Phenzopyradine | 19 (4) |
| NSAIDs | 10 (2.1) |
| Salicylate-containing product | 5 (1.1) |
| Probiotics | 4 (0.9) |
| Vaginal estrogen^c^ | 35 (11.7) |
| ^a^Denominator calculated among patients that had ≥ 1 medication listed (n=471) unless otherwise indicated  ^b^Includes anti-cholinergic and anti-muscarinic agents, alpha-blockers, and beta-3 adrenergic agonists  ^c^Denominator calculated from visits among females >50 years-old with ≥ 1 medication listed (n=298)  NSAID-non-steroidal anti-inflammatory | |
